# Incidence of proximal deep vein thrombosis in medical critical care patients

**DOI:** 10.1186/s12959-022-00363-5

**Published:** 2022-02-05

**Authors:** Chairat Permpikul, Walailak Chaiyasoot, Anupol Panitchote

**Affiliations:** 1 Division of Critical Care Medicine, Department of Medicine, Faculty of Medicine, Siriraj Hospital, Mahidol University, Bangkok, Thailand; 2 Interventional Radiology Unit, Department of Radiology, Faculty of Medicine, Siriraj Hospital, Mahidol University, Bangkok, Thailand; 3 Division of Critical Care Medicine, Department of Medicine, Faculty of Medicine, Khon Kaen University, Khon Kaen, Thailand

**Keywords:** Deep vein thrombosis, Critically ill patients, Prevalence, Incidence proportion, Ultrasonography

## Abstract

**Background:**

Approximately 13-31% of medical critical care patients develop deep vein thrombosis (DVT). However, there are very few reports regarding the incidence of DVT among Asian patients without routine prophylaxis. The objectives of this study were to assess the prevalence and incidence proportion of proximal DVT in Thai medical critical care patients not receiving thrombosis prophylaxis.

**Methods:**

We conducted a prospective cohort study in medical critical care patients admitted to Siriraj Hospital, Thailand between November 2008 and November 2009. Patients were screened for proximal DVT by duplex ultrasonography performed 48 h, 7, 14 and 28 days after admission. Primary outcomes were prevalence and incidence proportion of DVT. Factors associated with the development of proximal DVT were evaluated by multivariate analysis.

**Results:**

Of the 158 patients enrolled in the study, 25 had proximal DVT (15.8%). Nine patients (5.7%) had DVT on the first test at 48 h, while 10 (6.3%), 2 (1.3%) and 4 (2.5%) patients had developed DVT on days 7, 14, and 28, respectively. Thus, the prevalence at the beginning of the study was 5.7% (95%CI 2.6-10.5) and the incidence proportion was 10.1% (95%CI 5.9-15.9). The multivariate analysis showed that age (odds ratio [OR] per 1-year increase was 1.04, 95% confidence interval [CI] 1.01-1.07), female gender (OR 4.05, 95%CI 1.51-12.03), femoral venous catheter (OR 11.18, 95%CI 3.19-44.83), and the absence of platelet transfusion (OR 0.07, 95%CI 0.003-0.43) were associated with the development of proximal DVT. Patients with proximal DVT had a longer hospital length of stay (22 days [IQR 11-60] vs. 14 days [7-23], *p* = 0.03) and spent more time on mechanical ventilation (10 days (3.3-57) vs. 6 days (3-12), *p* = 0.053) than patients without DVT. Patient mortality was not affected by the presence of DVT (52% vs. 38.3%, *p* = 0.29).

**Conclusions:**

Routine thromboprophylaxis is not used in our institution and the prevalence and incidence proportion of proximal DVT in Asian medical critical care patients were both substantial. Patients with older age, female gender, an intravenous femoral catheter, and the absence of platelet transfusion all had a higher chance of developing proximal DVT.

## Background

Venous thromboembolism (VTE) is a life threatening clinical syndrome consisting of deep venous thrombosis (DVT) and pulmonary embolism [1]. This condition is not uncommon in critically ill patients as the risk factors include prolonged immobilization, massive trauma, vascular injury, indwelling venous catheter, malignancy, and sepsis [2]. In certain patients, VTE may be asymptomatic [3]. An acute deterioration of hemodynamic status and gas exchange kinetics in patients with preexisting critical illness can leads to much worse outcomes. Pharmacologic thromboprophylaxis reduces the incidence of VTE [4], while adjunctive treatment with intermittent pneumatic compression did not have additional benefits in proximal lower limb DVT prevention [5]. However, this prevention strategy has not been implemented in our unit due to the belief that the risk of VTE is low in Asian patients [6–9]. Therefore, we conducted a prospective study in our institution to better examine the prevalence of VTE in our medical critical care patients. Prevalence and incidence proportion of proximal DVT and the factors associated with DVT development were evaluated.

## Methods

### Study setting and patient population

A prospective observational study of critically ill patients admitted to the medical ICU and medical wards of Siriraj Hospital, Mahidol University, Bangkok, Thailand between November 1 2008 and November 30 2009. The inclusion criteria were: (1) age > 18 years, (2) acute respiratory failure requiring invasive mechanical ventilation, (3) acute kidney injury requiring renal replacement therapy, and (4) sepsis defined according to SIRS criteria (ACCP/SCCM consensus conference 1992) [10]. Postoperative patients, patients with major trauma, or spinal cord injuries, or burns, and patients receiving anticoagulant therapy were excluded. This study was approved by the Siriraj Institutional Review Board (Si 545/2008). All patients or their next of kin provided written informed consent.

### Data collection and definitions

Proximal DVT was diagnosed by compression venous ultrasonography using real-time B-mode on Philips/HP image point Hx® multimodality ultrasound system with a 7.5 MHz and/or a 5.5 MHz linear transducer. The examination was begun just below then inguinal ligament by placing a linear transducer in a transverse position across the common femoral vein (CFV). Downward pressure was applied to the linear transducer until the vein collapsed completely (as seen on a gray-scale image) and then the transducer was relaxed. Downward pressure and relaxation were applied in 1-cm increments along the thigh from the CFV to the popliteal vein [11]. The deep veins in the lower leg were not examined. The compression venous ultrasonography was applied to both sides. Proximal DVT was diagnosed as a lack of full compressibility; a vein was considered fully compressible if no residual lumen was seen [12]. Color flow and spectral wave Doppler analysis were used for confirmation of proximal DVT.

Compression venous ultrasonography was first performed within 48 h of patient enrollment, and was repeated on day 7, day 14, and day 28, or earlier if clinically indicated [13]. Prevalence of DVT was the percentage of DVT that were diagnosed within the first 48 h of study inclusion. Incidence of DVT was characterized by the number of DVT diagnosed after the initial 48 h tests [14, 15].

Compression ultrasonography with color Doppler imaging was performed by an experienced physician (Panitchote A) and recorded in a digital video disc that was reviewed by a radiologist (Chaiyasoot W). The radiologist did not know the test results and patient’s clinical history. Interobserver reliability, kappa index, between the operator and the radiologist was 1.0.

The clinical pretest probability for DVT using Well’s criteria [16] was evaluated during the first 24 h and on day 7, day 14, and day 28 of the study. The score was categorized into low, moderate, or high probability groups. Demographic data included age, sex, body mass index, admission diagnosis, severity of illness based on the acute physiology, age, chronic health evaluation (APACHE) II score, presence of malignancy, and a previous history of VTE. Treatments and interventions during the patient’s hospital stay, such as renal replacement therapy (RRT), mechanical ventilation, platelet transfusion, vasopressor and inotropic drugs, neuromuscular blocking agents, and central venous catheter were collected. Patient outcomes consisted of duration of RRT, duration of mechanical ventilation, mortality, and length of hospital stay. The study data were collected and managed using REDCap [17].

### Statistical analysis

The sample size was based on estimating a population proportion with an overall two-sided alpha level of 0.05 under the assumption that the incidence of DVT in critically ill patients would be 13%. Prevalence and incidence proportion of proximal DVT were described as count and percentage with 95% confidence interval (CI). The factors associated with proximal DVT were explored. Continuous factors are presented as mean (standard deviation) or median (interquartile range, IQR). Discrete factors are presented as count and percentage. Two-sample *t* test or Wilcoxon rank sum were used to compare continuous features, as appropriate. Chi square test or Fisher’s exact test were used to compare discrete variables, as appropriate. The associations of these factors a with development of proximal DVT were analyzed by a generalized linear regression with a binomial distribution and logit link function. To build a multivariable regression model, univariable regression was first performed. The features that were significant at *p* < 0.1 on univariable analysis were identified as potential predictor variables and entered into a multivariable regression model. Variable selection technique was performed using backward and forward stepwise model selection based on the Akaike information criterion. We checked multicollinearity in a final model using a variance inflation factor. Four-fold cross validation was used in estimating the generalization error. Area under the receiver operating characteristic (ROC) was calculated from four-fold cross validation for determination the model performance. We used R software version 3.5.2 for analysis (R Foundation for Statistical Computing, Vienna, Austria) [18]. The level of statistical significance was set at *p* < 0.05 (two tailed).

## Results

A total of 170 patients were enrolled in this study. Of this, 12 patients (7.1%) had inconclusive compression venous ultrasonography results and were excluded. Five patients had inconclusive results at day 14, four patients at day 7, two patients at day 2, and one patient at day 28. Thus, the remaining 158 patients were analyzed. Median patient age was 64.5 years (IQR 47-76). Seventy-six patients (48.1) were female. The main etiologies of ICU admission were acute respiratory failure (81.6% of the patients), followed by sepsis (78.5%), acute kidney injury (27.8%), congestive heart failure (7%), chronic obstructive pulmonary disease with exacerbation (6.3%), stroke (5.7%), and acute respiratory distress syndrome (5.7%). Mean APACHE II score was 21±7.5 points (Table 1).

**Table 1:** Clinical characteristics of all cohort patients

Characteristic	All cohort patients (*n*=158)	
Age, median (IQR), years	64.5	(47-76)
Female sex, n (%)	76	(48.1)
Body mass index, median (IQR), kg/m^2^	21.6	(19.1-24.2)
Acute conditions, n (%)		
Acute respiratory failure	129	(81.6)
Sepsis	124	(78.5)
Acute kidney injury	44	(27.8)
Congestive heart failure	11	(7)
COPD with acute exacerbation	10	(6.3)
Cerebrovascular accident	9	(5.7)
Acute respiratory distress syndrome	9	(5.7)
APACHE II, mean (SD), points	21	(7.5)
History of cancer, n (%)	25	(15.8)
History of previous VTE, n (%)	2	(1.3)
Renal replacement therapy (RRT), n (%)	25	(15.8)
Duration of RRT, median (IQR), days	3	(1-19)
Platelet transfusion, n (%)	28	(17.7)
Use of vasopressor or inotropes, n (%)	107	(67.7)
Mechanical ventilation, n (%)	133	(84.2)
Use of neuromuscular blocking agent, n (%)	24	(15.3)
Central venous line, n (%)	85	(53.8)
Internal jugular vein	73	(46.2)
Femoral vein	24	(15.2)
Subclavian vein	8	(5.1)
Cubital vein	9	(5.7)

APACHE = acute physiology, age, chronic health evaluation, ARDS = acute respiratory distress syndrome, COPD = chronic obstructive pulmonary disease, DVT = deep vein thrombosis, IQR = interquartile range, NMBA = neuromuscular blocking agent, RRT = renal replacement therapy, VTE = venous thromboembolism

Twenty-five patients (15.8%) developed proximal DVT at some point during the study. Nine of these (36.0%) had proximal DVT upon the first test within 48 h, while 10 patients (40%), 2 patients (8%), 4 patients (16%) were diagnosed at days 7, 14, and 28 respectively. Hence, the point prevalence of proximal DVT at the beginning of the study was 5.7% (95%CI 2.6-10.5) and the incidence proportion over 28 days was 10.1% (95%CI 5.9-15.9). Low clinical probability of DVT was found common (76.6%) inthe first 48 hours (Table 2). The clinical pretest probability of DVT at the first 48 h was significantly associated with ultrasound diagnosis (*p* = 0.02) and was a significant predictor of development of proximal DVT (*p* = 0.02).

**Table 2:** Clinical probability of DVT at screening time in all cohort patients

Clinical probability	Low		Moderate		High	
First 48 h, n (%)	121	(76.6)	29	(18.4)	8	(5.1)
Day 7, n (%)	52	(50)	40	(38.5)	12	(11.5)
Day 14, n (%)	31	(52.5)	25	(42.4)	3	(5.1)
Day 28, n (%)	16	(57.1)	11	(39.3)	1	(3.6)

The common femoral vein was the most common site of proximal DVT (21 patients [84%], 95%CI 63.9-95.5), followed by popliteal vein (8 patients [32%], 95%CI 14.9-53.5) and superficial femoral vein (7 patients [28%], 95%CI 12.1-49.4). In addition, 2 patients (8%) had bilateral DVT. Symptomatic proximal DVT occurred in 16 patients (64%) and sudden cardiac arrest suspected pulmonary embolism occurred in 1 patient.

### Factors associated with development of proximal DVT

Patient age and gender were both associated with diagnosis of proximal DVT. The median age of patients with a diagnosis of proximal DVT (72 years) was significantly higher than patients without proximal DVT (61 years, *p*=0.04), and a higher proportion of proximal DVT patients were female (68% vs. 44.4%, *p*=0.051). More patients with proximal DVT underwent renal replacement therapy (32%) and femoral venous catheterization for renal dialysis (36%) than patients without proximal DVT (12.8% and 1.3%, respectively). There was no association of proximal DVT with severity of illness, history of malignancy, receiving neuromuscular blocking agents, vasopressors, or mechanical ventilation (Table 3).

**Table 3:** Factors associated with proximal deep vein thrombosis

Characteristic	No DVT (133)	DVT (25)	*p*	Multivariable analysis^a^	
				Adjusted OR (95% CI)	*p*
Age, median (IQR), years	61 (45-75)	72 (64-80)	0.04	1.04^b^ (1.01-1.07)	0.02
Female sex, n (%)	59 (44.4)	17 (68)	0.051	4.05 (1.51-12.3)	0.01
BMI, median (IQR), kg/m^2^	21.6 (19-24.2)	21.3 (19.2-24.4)	0.83		
Acute conditions, n (%)					
Acute respiratory failure	107 (80.5)	22 (88)	0.57		
Sepsis	104 (78.2)	20 (80)	0.99		
Acute kidney injury	37 (27.8)	7 (28)	0.99		
Congestive heart failure	9 (6.8)	2 (8)	0.69		
COPD with acute exacerbation	8 (6)	2 (8)	0.66		
Cerebrovascular accident	9 (6.8)	0 (0)	0.36		
ARDS	8 (6)	1 (4)	0.99		
APACHE II, mean (SD), points	20.8 (7.7)	22.3 (5.7)	0.26		
History of cancer, n (%)	22 (16.5)	3 (12)	0.77		
History of previous VTE, n (%)	1 (0.8)	1 (4)	0.29		
RRT, n (%)	17 (12.8)	8 (32)	0.03		
Duration of RRT, median (IQR), days	2 (1-19)	6.5 (2.5-13.3)	0.68		
Platelet transfusion, n (%)	27 (20.3)	1 (4)	0.08	0.07 (0.003-0.43)	0.02
Use of vasopressor or inotropes, n (%)	89 (66.9)	18 (72)	0.79		
Use of NMBA, n (%)	18 (13.6)	6 (24)	0.22		
Central venous line, n (%)	68 (51.1)	17 (68)	0.18		
Internal jugular vein	59 (44.4)	14 (56)	0.94		
Femoral vein	15 (11.3)	9 (36)	0.004	11.18 (3.19-44.83)	< 0.001
Subclavian vein	5 (3.8)	3 (12)	0.11		
Cubital vein	8 (6)	1 (4)	0.99		
Mechanical ventilation, n (%)	111 (83.5)	22 (88)	0.77		
Clinical probability at first 48 h, n (%)					
Low	106 (79.7)	15 (60)	0.02		
Moderate	23 (17.3)	6 (24)			
High	4 (3)	4 (16)			

APACHE = acute physiology, age, chronic health evaluation, ARDS = acute respiratory distress syndrome, BMI = body mass index, CI = confidence interval, COPD = chronic obstructive pulmonary disease, DVT = deep vein thrombosis, IQR = interquartile range, NMBA = neuromuscular blocking agent, OR = odds ratio RRT = renal replacement therapy, VTE = venous thromboembolism

^a^Area under the receiver operating characteristic curve (95% CI) of multivariable analysis = 0.72 (95% CI 0.60–0.85)

^b^per 1 point increase

After multivariable analysis, development of proximal DVT was independently associated with age (OR 1.04, 95%CI 1.01-1.07, *p* = 0.02), female sex (OR 4.05, 95%CI 1.51-12.03, *p*=0.01), presence of femoral venous catheterization (OR 11.18, 95%CI 3.19-44.83, *p* < 0.001), and the absence of platelet transfusion (OR 0.07, 95%CI 0.003-0.43, *p* = 0.02) (Table 3). The area under the ROC of the multivariable model was 0.72 (95%CI 0.60-0.85).

### Proximal DVT and patient outcomes

Patients with proximal DVT had a higher duration of mechanical ventilation (10 [IQR 3.3-57] vs. 6 [IQR 3-12], *p* = 0.053) and a longer length of hospital stay (22 [IQR 11-60] vs. 14 [IQR 7-23], *p* =0.03). However, there was no association with hospital mortality (13 [52%] vs. 51 [38.3%], *p* = 0.29) (Table 4).

**Table 4:** Patient outcomes by proximal deep vein thrombosis

Outcome	All (*n*=158)	No DVT (*n*=133)	DVT (*n*=25)	*p*
Duration of MV, median (IQR), days	6 (3-13)	6 (3-12)	10 (3.3-57)	0.053
Hospital length of stay, median (IQR), days	14 (8-27)	14 (7-23)	22 (11-60)	0.03
Mortality, n (%)	64 (40.5)	51 (38.3)	13 (52)	0.29

DVT = deep vein thrombosis, IQR = interquartile range, MV = mechanical ventilation

## Discussion

In our medical ICU, where routine prevention of venous thromboembolism is not employed, the occurrence of proximal DVT was 15.8%. The prevalence at the beginning of the study was 5.7% while the incidence proportion during the first 28 days was 10.1%. The common femoral vein was the most common site, followed by the popliteal vein and the superficial femoral vein. Three-fourths of all DVTs developed within 7 days of the patients’ inclusion in the study. Age, female sex, presence of femoral venous catheterization and absence of platelet transfusion were significant factors associated with development of DVT. Patients who had proximal DVT also had longer hospital stays and tended to spend more time on mechanical ventilation. However, there was no association between DVT and patient mortality. Despite the fact that this study was conducted more than ten years before this report, we believe that the data are sound because our institution’s ICU practices regarding prevention and care of DVT remain unchanged.

The occurrence of proximal DVT in our MICU patients, who did not undergo thromboprophylaxis, is paralleled in other reports. Data collected from a Chinese ICU in 2008 [19], which focused on medical patients, disclosed a combined distal and proximal lower extremity DVT incidence of 19% and a proximal DVT incidence of 7.5%. However, this study excluded patients with a femoral venous catheter, which was one of the factors associated with the development of proximal DVT in our study. One systematic review revealed that the DVT incidence ranged from 13 to 31% [20–24]. It is noticeable that the prevalence of DVT in our patients was similar to rates reported in Chinese populations and less similar to rates reported in western (Caucasian) populations. DVT was also found in patients receiving thromboprophylaxis, as noted in a prospective study of medical – surgical critically ill patients. In this study, the prevalence of DVT on ICU admission (within 48-72 h) was 2.7% and incidence was 9.6% over the ICU stay [16]. Another study from China revealed similar findings with the incidence of VTE in sepsis patients receiving thromboprophylaxis 9.95% at 28 days [25]. Another prospective study [26] revealed an overall VTE incidence, which included lower and upper extremity thrombosis, of 37.2% and use of central venous catheter and mechanical ventilation were identified as significant risks. Our study population consisted of a large proportion of sepsis patients (78.2%), but the DVT incidence was not similarly high. The reasons might include the fact that our study did not include upper extremity examination and the ethnic differences between the studies. Therefore, our results fill the knowledge gap regarding DVT incidence in the Asian population.

Patient age, gender, presence of femoral venous catheter, and absence of platelet transfusion were all significant factors associated with the occurrence of thrombosis in our study. Age has been identified in several studies as a significant VTE risk [27–30]. Advancing age poses thrombotic risks because of increases in coagulation factors, platelet reactivity, and impairment of fibrinolytic activity [31]. As for the sex difference, while our study showed that being female was associated with developing proximal DVT, other studies have reported different results [32]. During childbearing years, the incidence rates of VTE are higher in females but the rates after age 45 years are higher in males [33]. Having a central venous catheter in place is a well-known DVT risk [34–36]. The femoral site poses a greater risk than subclavian locations [37] while the DVT rate of internal jugular veins is similar [38]. Blood transfusions are associated with DVT, particularly red blood cell and/or fresh frozen plasma transfusion but platelet transfusion is not associated with DVT [39]. However, the study of Cook showed that platelet transfusion is associated with DVT [14]. Although our study showed the opposite result, all the patients with platelet transfusion had thrombocytopenia as an indication for platelet transfusion. It might imply that thrombocytopenia may be a preventive factor for the development of DVT. However, future studies need to be answered.

Our study showed that patients with DVT had prolonged hospital stays and showed a marginal increase in time spent on mechanical ventilation while there was no association between DVT and patient mortality. However, the statistical power of this study for detection of mortality differences is insufficient. Our study was in line with the studies of Cook and Malato [14, 40].

We acknowledge a number of limitations in the current study. We did not confirm diagnosis of DVT by venography because it is difficult to perform in critically ill patients. Duplex ultrasonography is operator dependent, so to minimize bias, all studied were recorded and reviewed by a blinded radiologist. We had some inconclusive ultrasonography results because of edema in the lower extremities. The considerable number of inconclusive results could lead to underestimating the incidence. Moreover, our study did not systemically focus on pulmonary embolism except that patient developed clinically suspected pulmonary embolism. Future studies about the incidence of both DVT and pulmonary embolism will be needed.

## Conclusions

The incidence proportion of proximal lower limb DVT among Asian medical critical care patients without thromboprophylaxis was slightly lower than reported for Caucasian populations. Patients at higher risks for development of proximal DVT were those with increased age, female gender, receiving a femoral venous catheter, and the absence of platelet transfusion. Patients with DVT stayed longer in hospital but did not show increased mortality. Based on our results, we would suggest routinely pharmacologic VTE prophylaxis in critically ill patients particularly a patient who is an elderly female and has a femoral venous catheter because the probability for development of DVT is more than 50%.

## Data Availability

The datasets used and/or analyzed during the current study are available from the corresponding author on reasonable request.

## Abbreviations

APACHE: acute physiology, age, chronic health evaluation
CI: confidence interval
CFV: common femoral vein
CUS: compression venous ultrasonography
DVT: deep vein thrombosis
ICU: intensive care unit
IQR: interquartile range
OR: odds ratio
ROC: the receiver operating characteristic
RRT: renal replacement therapy
VTE: venous thromboembolism
